# Wigner Crystal and Colossal Magnetoresistance in InSb Doped with Mn

**DOI:** 10.1038/srep13451

**Published:** 2015-08-26

**Authors:** S. A. Obukhov, S. W. Tozer, W. A. Coniglio

**Affiliations:** 1 Department of Solid State Electronics, Ioffe Physical-Technical Institute of the Russian Academy of Sciences, St Petersburg 194021, Russian Federation; 2 National High Magnetic Field Laboratory, Florida State University, Tallahassee 32310, USA

## Abstract

We report magnetotransport investigation of nonmagnetic InSb single crystal doped with manganese at Mn concentration N_Mn_ ~ 1,5 × 10^17^ cm^−3^ in the temperature range T = 300 K–40 mK, magnetic field B = 0–25T and hydrostatic pressure P = 0–17 kbar. Resistivity saturation was observed in the absence of magnetic field at temperatures below 200 mK while applied increasing external magnetic field induced colossal drop of resistivity (by factor 10^4^) at B ~ 4T with further gigantic resistivity increase (by factor 10^4^) at 15T. Under pressure, P = 17 kbar, resistivity saturation temperature increased up to 1,2 K. Existing models are discussed in attempt to explain resistivity saturation, dramatic influence of magnetic field and pressure on resistivity with the focus on possible manifestation of three dimensional Wigner crystal formed in InSb by light electrons and heavy holes.

A crystalline state of low density electron gas was predicted by Wigner^1^ and experimentally observed in one and two dimensional structures^2^,^3^ and bulk magnetic semiconductor Hg_1−v−x_Cd_v_Fe_x_Se with x ≤ 0,15 [Ref. 4]. Halperin and Rice^5^ put forward the idea that semiconductors with electrons at low density and large difference in effective masses between electrons and holes could be the appropriate environment for the realization of Wigner crystallization in semiconductors. It was proposed that in this type of system electrons and holes could be held together by Coulomb attraction and thus form excitons, which can condensate and create a periodic array of excitonic molecules analogous to solid hydrogen. In this condensate heavy holes can form a crystal in which electron movement resembles the electron motion in metals. In this sense InSb crystal having a large hole/electron masses ratio (*m*_h_/*m*_e_ = 0,5/0,01 ~ 50) can be a possible candidate to form 3D Wigner crystal. In case the concentration of electrons in crystal is below n_e_ < 10^14^ cm^−3^, InSb parameters will be close to Wigner crystallization criteria, i.e. n_e_^1/3^a_B_ < 1 [Ref. 5] and Bohr electron radius of about 600A [Ref. 6].

Our previous magnetotransport studies of InSb crystals doped with manganese^7^,^8^,^9^,^10^ revealed that electrons and holes interplay modified InSb(Mn) conductivity at low temperatures and brought about unusual resistivity- temperature dependence (ρ-T) at manganese concentration of about N_Mn_ ~ 10^17^ cm^−3^. We also observed the dramatic resistivity increase under pressure (ρ_P_/ρ_P=0_ ~ 10^5^ at P = 15 kbar and T ~ 1,5 K [Ref. 9] and simultaneous decrease in electrons and holes concentration which suggests that formation of electron-hole (e-h) insulating pairs or permanent (metastable) excitons can account for the unexpected InSb(Mn) behavior. This observation is in accordance with suggested features of the Wigner crystal which are conductivity metallization and low electrons concentration^5^. The main goal of the presented study is to verify if InSb could be an appropriate material for formation and study of Wigner crystal phase. As temperature and pressure are the critical parameters for Wigner crystallization we performed magnetotransport studies of InSb(Mn) crystal within the millikelvin temperature range and pressure up to 17 kbar.

## Results and Discussion

### Temperature-dependent resistivity

**Figure 1:**
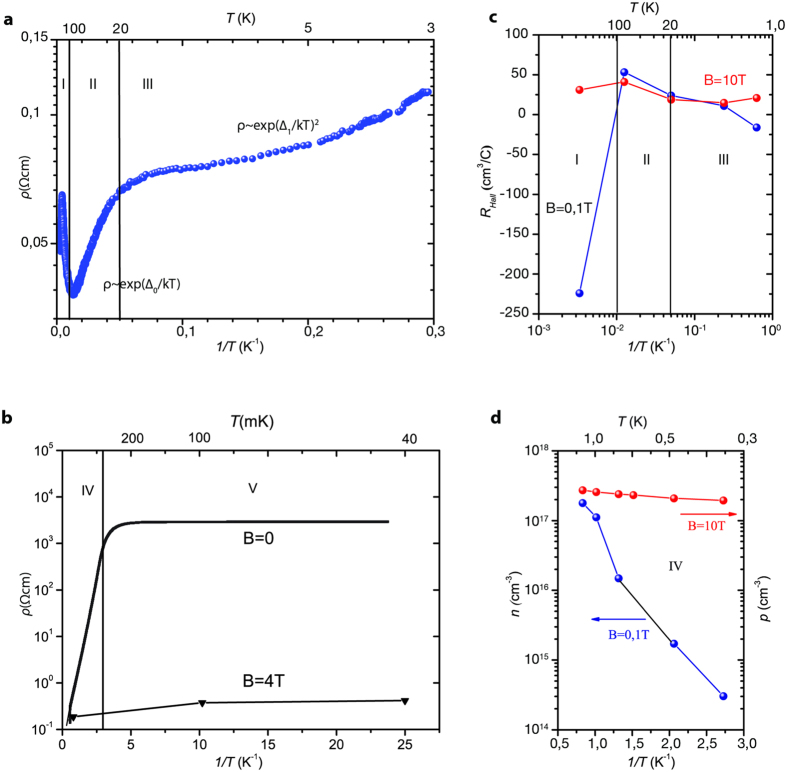
Specific resistivity, the Hall constant and charge carriers concentration as a function of 1/T at P = 0 kbar in p-InSb(Mn) sample at N_Mn_ = 1,6 × 10^17^ cm^−3^. (**a**) Temperature- resistivity dependence of p-InSb(Mn). The vertical solid lines mark the temperature ranges **I**- (T = 300 ÷ 80 K), **II**- (T = 80 ÷ 20 K) and **III**- (T = 10 ÷ 1,5 K). (**b**) Specific resistivity as a function of temperature in the millikelvin temperature range at B = 0 and B = 4T (temperature ranges **IV** at 1,5 ÷ 0,3 K and **V** at 0,3 K ÷ 40 mK). (**c**) The Hall constant R_H_ as a function of temperature at B = 0,1T and B = 10T (temperature range 300 ÷ 1,5 K). (**d**) Electrons and holes concentration as a function of temperature below 1,5 K at B = 0,1T and B = 10T. Concentration of electrons and holes were estimated as n, p = 6,25 × 10^18^/R_H_.

Figure 1a,b show the resistivity—temperature dependence for p-InSb(Mn) crystals at N_Mn_ = 1,6 × 10^17^ cm^−3^ and ambient pressure. The graphs clearly demonstrate that changes in resistivity-temperature dependence can be diverse within five different temperature ranges (I–V):

(I) 300 ÷ 80 K—with the sharp rise of resistivity to its maximum at T ~ 250 K (ρ = 0,065 Ωcm), further drop of resistivity and final resistivity minimum at T = 80 K (ρ = 0,037 Ωcm).

(II) 80 ÷ 20 K—is characterized by exponential increase of resistivity


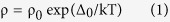


where Δ_0_ = 1,72 meV and ρ_0_ = 0,03 Ωcm.

(III) 10 ÷ 1,5 K—within this range resistivity changes can be described with quadratic exponential expression^7^


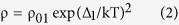


at Δ_1_ = 0,2 meV and ρ_01_ = 0,07 Ωcm.

(IV) 1,5 ÷ 0,3 K—here resistivity exponentially increases


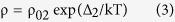


at Δ_2_ = 0,28 meV and ρ_02_ = 0,04 Ωcm.

In contrast to above results for semiconductors doped with nonmagnetic shallow acceptors like p-Ge(Ga) and p-Si(B) crystals resistivity-temperature dependence within the temperature range 1,5–0,3 K is usually described in the framework of hopping conductivity models


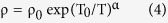


where T_0_ and ρ_0_ are temperature independent constants, α = 0,25 and α = 0,5 are indexes of Mott conductivity^11^ and Coulomb gap model^12^ respectively.

(V) 200 ÷ 40 mK—the range where resistivity is independent of temperature at ρ_sat_ = 3 × 10^3^ Ωcm. This metallization of conductivity below 200 mK, which we observed in p-InSb(Mn) crystal, was dramatically different from the low temperature hopping conductivity usually revealed in doped semiconductors (Eq. 4). According to the model of exitonic insulator^5^ we can view the resistivity saturation ρ_sat_ at T < 200 mK as one of Wigner crystal conductivity characteristics where T_cryst_ = 200 mK should be denoted as the temperature of Wigner crystallization.

### The Hall effect

To get information about charge carriers and their concentration we studied the Hall constant dependence on temperature and magnetic field at ambient pressure (P ≈ 0 kbar). It was revealed that the Hall constant can change its sign under variable temperature and magnetic field values as it is shown in Fig. 1c where R_H_-T dependence is presented within the temperature ranges (**I** ÷ **V**) observed for ρ-T dependence:

- 300 ÷ 80 K—R_H_ is negative in low magnetic field (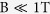, T ≈ 300 K) while it is positive in high magnetic field (B > 1T, T = 300 K). Such behavior of R_H_ as a function of magnetic field is typical for semiconductors with two charge carriers– electrons with small effective mass and heavy holes^6^.
- 80 ÷ 20 K—where the hole conduction dominates, the Hall coefficient is positive and becomes magnetic field independent. Conductivity in this temperature range is determined by the gap energy Δ_0_ = 1,72 meV between the valence and the impurity bands (Eq. 1). We note that in InSb an acceptor activation energy of manganese is E_a_ = E_Mn_ = 9 meV [Ref. 6].
- 10 ÷ 1,5 K—here we again observe mixed conductivity like conductivity at ambient temperature. In this case R_H_ is negative for the low magnetic field and positive for the high magnetic field, which means that at low temperatures in p-type p- InSb(Mn) crystals the intrinsic electrons conduction dominates. We highlight that mixed conduction at low temperatures is a specific feature of InSb crystals doped with Mn. Neither InSb crystals doped with germanium- p-InSb(Ge) [Ref. 9] nor p-Ge(Ga) [Ref. 13] and p-Si(B) [Ref. 14], demonstrate n-type conductivity at low temperatures. The above observations of R_H_-B dependence in p-InSb(Mn) have given us possibility to relate the resistivity-temperature dependence described by Eq. 2 (T = 10 ÷ 1,5 K) to the input of e-h insulating pairs, or “permanent” excitons, into conductivity^15^,^16^. It results in formation of permanent excitons extracting charge carries from the conductive crystal, the process which leads to the quadratic exponential increase of resistivity as temperature declines.
- 1,5 K ÷ 0,3 K—this temperature range is associated with the decrease of electrons and holes concentration resulting in active formation of excitonic insulator phase (Fig. 1d).
- 200 ÷ 40 mK—the tendency towards the decrease of charge carrier concentration can be clearly seen from the Hall effect measurements in the range 1,5 K–0,3 K (Fig. 1d), but we were not able to get information about electrons and holes concentration at low magnetic field due to the high sample resistance.

### Pressure influence on transport properties

**Figure 2:**
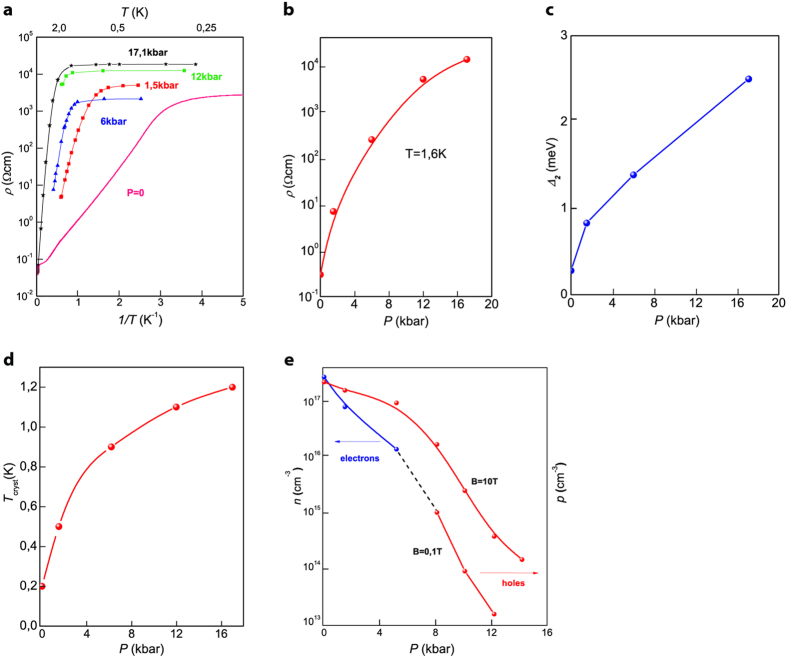
Resistivity and charge carriers concentration under hydrostatic pressure in InSb(Mn). (**a**) ρ-T dependence in p-InSb(Mn) at various pressure regimes. (**b**) Resistivity as a function of pressure at T = 1,6 K. (**c**) Activation conduction energy Δ_2_. (**d**) Wigner’s crystallization temperature T_cryst_. (**e**) Charge carriers concentration vs pressure—n and p values revealed from the Hall effect measurements at B = 0,1T and B = 10T at T = 1,6 K. The dash curve indicates the transition from electron to hole conduction under pressure.

Resistivity-pressure dependence of p-InSb(Mn) crystal is shown in Fig. 2a, which demonstrates that pressure induces specific resistivity increase within the whole range of temperatures below 4 K (Fig. 2a,b). We should highlight two observations arising from ρ-T dependence investigation as a function of pressure. Firstly, within the activated conductivity range (above T_cryst._) resistance increase under pressure is induced by higher activation conduction energy Δ_2_ (Fig. 2c). Secondly, under pressure crystallization temperature T_cryst_ shifts towards the higher temperature range and reaches T = 1,2 K at P = 17 kbar (Fig. 2d).

The Hall constant measurements at T = 1,6 K revealed that under hydrostatic pressure between P = 5 kbar and 8 kbar the conduction electrons “vanish” (Fig. 2e). We suggest that such behavior of the Hall constant is the result of permanent (metastable) excitonic insulator phase formation. We also propose that pressure stimulates increase of electron crystallization temperature and thus shifts T_cryst_ to higher levels (Fig. 2d). The similar concept of pressure induced excitonic insulator was introduced for pressure-driven semiconductor-metal transition in single-crystalline TmSe_1−x_Te_x_ [Ref. 17].

The artificial solid formed in InSb(Mn) in the proposed above Wigner crystallization model differs from the Wigner crystal structure discussed for Hg_1−v−x_Cd_v_Fe_x_Se with x < 0.l5 and v ≤ 0,4, where Fe^3+^ (3*d*^5^) are ionized donors used for “charge superlattice” formation^4^. By contrast, according to the presented here Wigner model light electrons form 3D lattice pinned with heavy holes, but not impurity ions, thus forming crystalline excitonium^5^,^18^. This conclusion results from the Hall effect measurements where we observed both electrons and holes concentration decrease under cooling and pressure. We propose also that electrons in Wigner crystal at low concentration form antiferromagnetically ordered phase exhibiting insulator behavior.

### Colossal Magnetoresistance

**Figure 3:**
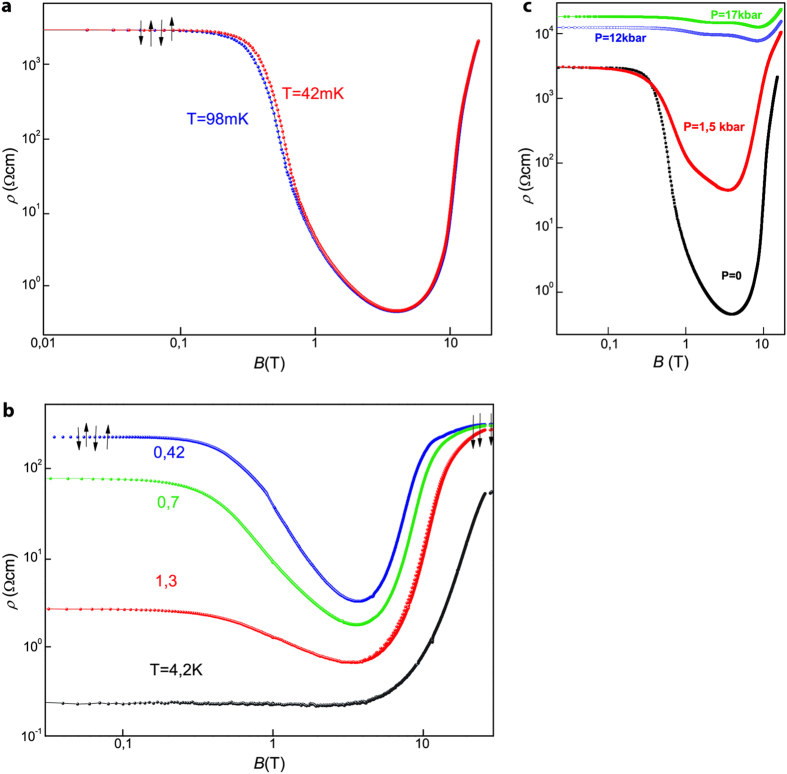
Colossal magnetoresistance in InSb(Mn). (**a**) Specific resistivity in p-InSb(Mn) crystal as a function of magnetic field at temperature below T_cryst_ at P = 0 kbar. Arrows show antiferromagnetic orientation of electron spins at B = 0 ÷ 0,1T. (**b**) CMR in InSb(Mn) at T > T_cryst_ in magnetic field up to 25T at P = 0 kbar. Arrows show antiferromagnetic orientation of electron spins at B < 0,1T and ferromagnetic orientation at B > 20T. (**c**) ρ-B dependence at set of pressure P = 0; 1,5; 12 and 17 kbar at T = T_cryst_.

The results of transverse magnetoresistance measurements within millikelvine temperature range show that resistance decreases under magnetic field, reaches minimum at B ~ 4T and then goes up with the further increase of magnetic field (Fig. 3a). Resistance first decreases under magnetic field, reaches its minimum at B ~ 4T and then goes up with higher magnetic field (Fig. 3a). Thus magnetic field of about 4T induces transition from the insulator to quasimetal conductivity type (Fig. 1b). CMR value remains practically independent of temperature at millikelvin temperature range below 100 mK (Fig. 3a). As temperature goes above T_cryst_ CMR decreases and disappears at temperature below 4 K. Above B = 15T resistivity saturates (Fig. 3b) which we relate to electrons transition to ferromagnetic order. As temperature goes above T_cryst_ CMR decreases and disappears at T = 4 K. Maximal values of CMR in p-InSb(Mn) in millikelvin temperature range is comparable with CMR previously observed in both Magnetic and Diluted Magnetic Semiconductors (MS and DMS) like Gd_1−x_v_x_S_4_ [Ref. 19] and Hg_1−x_Mn_x_Te [Ref. 20] as well as in Manganite Perovskite Pr_0,7_Ca_0,26_Sr_0,04_MnO_3_ [Ref. 21]. Important observation is that manganese concentration in InSb(Mn) was smaller by a factor 10^4^ ÷ 10^5^ compared to magnetic ions concentration in MS, DMS and Manganite Perovskites.

Though a great many models have been meanwhile proposed for the explanation of CMR in *magnetic* and *nonmagnetic* materials the following seem to be of particular interest:

1. Phase separation model^22^ developed for hole–doped manganese oxides describing CMR as induced by magnetic field transition from antiferromagnetic insulator phase to conducting ferromagnetic state.
2. Magnetic polaron model^23^ proposed for MS explains declining resistance in magnetic field as the decrease in charge carries scattering on magnetic clusters.
3. The model proposed for DMS Hg_1−x_Mn_x_Te connected with dramatic change in hole effective mass in magnetic field^24^.
4. Zeeman splitting model for nonmagnetic Ge crystal doped with Cu [Ref. 25].
5. Excitonic insulator (EI) model trying to describe the effect of transition from excitonic insulator phase to conducting state induced by external magnetic field and adapted to CMR in *nonmagnetic* InSb crystals doped with manganese^8^.

It is still hard to adapt above models for MS and DMS in the case of InSb crystals doped with manganese at impurity concentration within N_cr_ = p = N_Mn_ = 2 × 10^17^ cm^−3^ as InSb is *nonmagnetic* semiconductor in contrast to *magnetic* materials where magnetic ions concentration is about 10^5^ times as large as N_cr_ for InSb(Mn). On the other hand, InSb gives us the unique possibility to compare the behavior of *magnetic* Mn—and *nonmagnetic* Ge- impurities, as Ge like Mn forms shallow acceptor level with the same energy at E_a_ = 9 meV^6^ and demonstrates exactly the same critical concentration of metal-insulator transition at N_cr_ = 2 × 10^17^ cm^−3^. But transport and magnetotransport characteristics of p-InSb(Ge) crystals at low temperatures radically differ from p-InSb(Mn) characteristics^7^. Crystals of p-InSb(Ge) do not demonstrate any resistivity features dependent on impurity concentration, temperature and magnetic field. Similar to p-Ge(Ga) and p-Si(B) the crystals of p-InSb(Ge) demonstrate the Variable Range Hopping conductivity, the ordinary Hall effect and positive magneotresistance typical for disordered localized *nonmagnetic* impurities. The difference in behavior of *magnetic* (Mn) and *nonmagnetic* (Ge) impurities in InSb could be related to the influence of Jahn-Teller (JT) lattice distortion caused by impurity manganese^26^. It was previously revealed that under uniaxial stress X ~ 3 kbar at T ~ 1,5 K both p-Ge(Ga) and p-InSb(Ge) demonstrate the decrease in specific resistivity by a factor 10^2^ in magnetic field B = 3-5T^27^. In p-InSb(Mn) crystals CMR also increased under uniaxial stress by a factor 10^4^ at T = 1,2 K^28^. Thus we can consider an uniaxiall stress in p-InSb(Ge) as a method to simulate JT distortion described by Bir^29^. The analogy between CMR in uniaxially stressed p-InSb(Ge) and unstressed p-InSb(Mn) crystals supports the idea of Jahn-Teller (JT) lattice distortion input in CMR. Both the uniaxial stress and the JT distortion in InSb(Mn) crystals splits the valence band and removes valence band degeneration boosting CMR effect.

Positive Magnetoresistance (PMR) observed at B > 4T could be also related to ferromagnetic orientation of electron spin, but alternative approach is the Anderson localization of electrons in high magnetic field. In this model PMR is the result of electron wave function diamagnetic shrinking^30^. On the other hand we revealed colossal decrease of resistivity in p-InSb(Mn) in magnetic field (CMR) between B = 0 and 4T at temperature below T_cryst_. In observation of resistivity saturation at low and superlow temperatures the lack of PMR in the system at low magnetic field and T < T_cryst_ is the point in the favour of the Wigner crystallization concept rather than of electron localization disorder.

Contrary to uniaxial stress hydrostatic pressure caused the decrease of CMR in InSb(Mn). The influence of pressure on magnetoresistance is shown in Fig. 3c. It can be seen that ρ at B = 4T decreased only by a factor 10^2^ at P = 1,5 kbar and at P > 14 kbar the influence of magnetic field on the resistance was insignificant.

In conclusion, revealed resistivity saturation, decrease in holes and electrons concentration at low temperatures, dramatic influence of magnetic field and hydrostatic pressure on resistivity we associate with the manifestation of the three-dimensional Wigner crystal formed in InSb(Mn) crystal. We propose that an external magnetic field at B ~ 4T and T ≤ T_cryst_ induces CMR effect as the transition from antiferromagnetic insulator state of electrons to metal state and induces metastable excitons dissociation at temperature above T_cryst_.

## Methods

InSb manganese doped single crystal was grown by Czochralsky method. Dimensions of the crystal was about 3 cm in diameter and 5 cm long. Impurity concentration of Mn varies along the crystal length from N_Mn_ ~ 10^17^ cm^−3^ at one end to the 4 × 10^17^ cm^−3^ at another end of the crystal. Samples for measurements were about 3 × 1 × 0,4 mm^3^ for measurements in the temperature range T = 300 ÷ 1,5 K and 0,3 × 0,2 × 0,12 mm^3^ for measurements in pressure cell at ^3^He cryostat and ^3^He–^4^He dilution refrigerator cryostat. Electrical conducting wires of gold were soldered to the samples with pure indium. Nonmagnetic pressure cells made of bronze provided hydrostatic pressure up to 16 ~ 17 kbar at low and super-low temperatures. The resistivity and the Hall effect at temperature ranges 300–1,6 K were measured in van der Pauw or the Hall bar geometry. The Hall resistance and transverse magnetoresistance measurements were carried out on the Hall bar samples in dilution refrigerator with 15T Oxford Instruments superconductive (SC) magnet at NHMFL, Florida. In other presented experiments with 10T and 18T Oxford Instruments SC magnets and 30T Bitter magnet of HMFL Nijmegen were used. Special attention was paid to magnetic field calibration of SC magnet used in experiments in millikelvin temperature range. Frequency measurements were done to detect ^1^H and ^3^He NMR lines, providing accurate field calibration data. Measured at 11 Tesla, repeatability in the same sweep direction was more than 5 mT. Repeatability when changing sweep direction was better than 20 mT. Absolute calibration at full field was within 1 mT on the downsweep, and field was 10 mT lower than displayed on the upsweep. The typical hysteresis of this magnet is less than 150 Oe. The lock-in measurements were carried out at the rate of 14 mT s^−1^. Zero magnetic field came to equilibrium when sweep was stopped. The sample was submerged in the cold ^3^He–^4^He mixture liquid, to exclude radiation factor.

## Additional Information

**How to cite this article**: Obukhov, S. A. *et al.* Wigner Crystal and Colossal Magnetoresistance in InSb Doped with Mn. *Sci. Rep.* **5**, 13451; doi: 10.1038/srep13451 (2015).

## References

[b1] Wigner E. On the interaction of electrons in metals. Phys. Rev. 46, 1002 (1934).

[b2] Kristinsd óttir L. H. *et al.* Signatures of Wigner localization in epitaxially grown nanowires. Phys. Rev. B 83, 041101(R) (2011).

[b3] Grimes C. C. & Adams G. Evidence for a liquid-to-crystal phase transition in a classical, two-dimensional sheet of electrons. Phys. Rev. Lett. 42, 795 (1979).

[b4] Mycieiski A. Fe- based semimagnelic semiconductors. J. Appl. Phys. 63, 3279 (1988).

[b5] Halperin B. I. & Rice T. M. Possible anomalies at a semimetal-semiconductor transition. Rev. Mod. Phys. 40, 755 (1968).

[b6] Bimberg D. Semiconductors (Springer, Berlin, 1982).

[b7] Obukhov S.A. Transport and magnetotransport effects in indium antimonide doped with manganese within quasimetal-insulator transition. Phys. Status Solidi C 9, 247 (2012).

[b8] Obukhov S.A. A new type of low temperature conductivity in InSb doped with Mn. AIP Advances 2, 022116 (2012).

[b9] Obukhov S.A. in Indium: Properties, Technological Applications and Health Issues. 81–122 (Novapublisher, New York, 2013).

[b10] Teubert J. *et al.* Influence of magnetic dopants on the metal-insulator transition in semiconductors. Phys. Rev. Lett. 102, 046404 (2009). 19257448 10.1103/PhysRevLett.102.046404

[b11] Mott N. F. Conduction in non-crystalline materials. Phil. Mag. 19, 835 (1969).

[b12] Shklovskii B. I. & Efros A. L. Electronic properties of doped semiconductors (Springer Verlag, Berlin-Heidelberg-New York-Tokyo, 1984).

[b13] Ftitzsche H. & Cuevas M. Impurity conduction in transmutation-doped p- type germanium. Phys. Rev. 119, 1238 (1960).

[b14] Alexander M. N. & Holcomb D. F. Semiconductor-to-metal transition in *n*-type group IV semiconductors. Rev. Mod. Phys. 40, 815 (1968).

[b15] Moskalenko S. A. & Snoke D. W. Bose—Einstein condensation of excitons and biexcitons. (University Press, Cambridge 2000).

[b16] Neueschwander J. & Wachter P. Pressure-driven semiconductor-metal transition in intermediate-valence TmSe_1−x_Te_x_ and the concept of an excitonic insulator. Phys. Rev. B 41 12693 (1990). 10.1103/physrevb.41.12693 9993746

[b17] Mycielski A. Fe-based semimagnetic semiconductors. Appl. Phys. 63, 3279 (1988). 10.1103/physrevb.53.10732 9982640

[b18] Abricosov A.A. Possible mechanism of high-temperature superconductivity. JETP Lett. 27, 219 (1978).

[b19] von Molnar S. & Holtzberg F. in Magnetism and Magnetic Materials. 1259–1273 (AIP Conference Proceedings, New York, 1973).

[b20] Wojtowicz T. & Mycielski A. An unusual magnetic field dependence of the acceptor ionization energy p-Hg_1−x_Mn_x_Te. Acta Physica Polonica A67, 363 (1985).

[b21] Raveau B. Les oxydes des metaux de transition des materiaux fonctionnels tres prometteurs. La lettre de l’Academie des sciences. (in French) 10, 8 (2003).

[b22] Moreo A, Yonoki S. & Dagotto E. Phase separation scenario for manganese oxides and related materials. Science 283, 2034 (1999). 10092219 10.1126/science.283.5410.2034

[b23] Coey J. M. D., Viret M. & von Molnar S. Mixed-valence manganites. Advances in Physics 48, 167 (1999).

[b24] Furdyna J. K. & Kossut J. in Semiconductors and Semimetals Diluted Magnetic Semiconductors. 25 (Academic Press, Inc. New York – London – Tokyo – Toronto, 1988).

[b25] Ganichev S. D. *et al.* Giant negative magnetoresistance in semiconductors doped by multiply charged deep impurities. Phys. Rev. B 63, 201204 (2001).

[b26] Jahn H. & Teller E. Stability of polyatomic molecules in degenerate electronic states. I. Orbital degeneracy. Proc. Royal Society London 161, 1934 (1937).

[b27] Obukhov S. A. Giant negative magnetoresistance in uniaxially stressed p-Ge and p-InSb single crystals. Phys. Status Solidi B 223, 535 (2001).

[b28] Averkiev N. S., Gey W., Obukhov S. A. & Rogachev A. A. Giant negative magnetoresistance in uniaxially deformed, manganese-doped indium antimonide. JETP Lett. 40, 773 (1984).

[b29] Bir G. L. Jahn-Teller effect on impurity centers in semiconductors. JETP 51, 556 (1966).

[b30] Ferre D., Dubois H. & Biskupski G. Structure of the impurity band and magnetic-field- induced metal-nonmetal transition in n-type InSb. Phys. Status Solidi B 70, 81 (1975).

